# Supplementary material for: Phosphatidylserine enrichment in the nuclear membrane regulates key enzymes of phosphatidylcholine synthesis
Source: EMBO J. 2024 Jun 25;43(16):3414–49. doi: 10.1038/s44318-024-00151-z (PMC11329639; doi:10.1038/s44318-024-00151-z)
Supplement: Supplementary file 1 — Table EV1 [file 44318_2024_151_MOESM1_ESM.doc]

Table EV1. Sequences of oligonucleotides, Related to Methods.

| CAG-NLS-myc-PSD1 F | ccgctcgagtattcgccacc**atggcttcttctggtcctattcgaactttacataagggtaaagccgcgcgaaatcgcacaccttacgatcgtattgca**ggcggagtgagcaaggagcagaagctgatctccgaggaagacctgttcggtggcggtg |
| --- | --- |
| CAG-2NES-myc-yPSD1 F | ccgctcgagtattcgccacc**atggctctgcagaaaaagttggaagagcttgagctggatgaggcaggagtggctctgcagaaaaagttggaagagcttgagctggatgaggca**ggagtgagcaaggagcagaagctgatctccgaggaagacctgttcggtggcggtg |
| CAG-myc-yPSD1 R | ataagaatgcggccgctcacttcaggtcgttcttg |
| CAG-myc-yPSD1 w/o EGFP F | tttggcaaagaattcctcgag |
| CAG-myc-yPSD1 w/o EGFP R | tactgtacatcacttcaggtcgttcttg |
| Cytosol-mCherry F | gacaccggtcgccaccatggtgagcaagggcgaggag |
| NLS-mCherry F | gacaccggtcgccacc**atgtcttctggtcctattcgaactttacataagggtaaagccgcgcgaaatcgcacaccttacgatcgtattgca**ggcgtgagcaagggcgaggag |
| NLS-mCherry-Lact^C2^ R | atctccggacttgtacagctcgtccatg |
| NLS/cytosol-mCherry-Evt^2xPH^ R | ctagatctgagtccggccggacttgtacagctcgtccatg |
| NLSmCherryLact^C2,AAA^ F1 | gtcagatccgctagcgctac |
| NLSmCherryLact^C2,AAA^ R1 | gtagtagggagccgcgctaaaggcactcaggcccgcggttttg |
| NLSmCherryLact^C2,AAA^ F2 | caaaaccgcgggcctgagtgcctttagcgcggctccctactac |
| NLSmCherryLact^C2,AAA^ R2 | gtaccgtcgactgcagaattc |
| single Evt^PH^ WT F | ggccggactcagatctcgatg |
| single Evt^PH^ WT R | atggtaccgtcgacg |
| single Evt^PH,K20E^ F1 | gacgagctgtacaagtc |
| single Evt^PH,K20E^ R1 | caaaccagttcttctcccagcg |
| single Evt^PH,K20E^ F2 | cgctgggagaagaactggtttg |
| single Evt^PH,K20E^ R2 | gatcccgggcccgcggtac |
| NLS-mCherry-Evt^PH,K20E^ F | cggggtaccatggcgtttgtgaagagtg |
| NLS-mCherry-Evt^PH,K20E^ R | gcggatccttagtttgtcctagaatcttg |
| ER^Lum^-mCherry F | gacaccggtcgccaccatggtg**aagctctccctggtggccgcgatgctgctgctgctcagcgcggcgcgggcc**gtgagcaagggcgaggaggataacatg |
| ER^Lum^-mCherry-Lact^C2^ R | cggaattccta**tagctcgtcttt**acagcccagcagctccactcgcag |
| ER^Lum^-mCherry-Evt^2xPH^ R | gatccggtgcggatcccta**tagctcgtcttt**gtttgtcctagaatcttg |
| HaloTag-Emerin F | ctagctagcgctaccggtcgccaccatggcagaaatcggtactg |
| HaloTag-Emerin R | atctccggagccggaaatctcgagcgtc |
| PSS1-HaloTag F | gcggatccaccggccggtcgccaccatggcagaaatcggtac |
| PSS1-HaloTag R | ataagaatgcggccgctttagccggaaatctcgagcgtc |
| CCTα(1-255) F | cccaagcttcgatggatgcacagtgttc |
| CCTα(1-255) R | gcggatccttaatctttcactttcttctttac |
| CCTα(1-255)-4hQ R | gcggatccttaatctttctgtttcttcttttgtttgtcttgcctctcctgctggtggtatttc |
| CCTα(1-255)-CLLL R | gcggatcctta**caacagcaggca**atctttcactttcttctttac |
| CCTα(1-255)-4hQ-CLLL R | gcggatcctta**caacagcaggca**atctttctgtttc |
| CCTα(1-255)-12Q-CLLL R | gcggatcctta**caacagcaggca**atcttgctgttgcttctgttgttggtcttgctgctcctgctgctggtattgctgctcgttg |
| CCTα-8pQ-FL F | cgagcagcaataccagttgcaggagcaggttgaccaagtacagaagcaagtgcaagatgtg |
| CCTα-8pQ-FL R | catcttgcacttgcttctgtacttggtcaacctgctcctgcaactggtattgctgctcgttg |
| CCTα(1-255)-8pQ-CLLL R | gcggatcctta**caacagcaggca**atcttgcacttgcttc |
| CCTα-8pA-FL F | cgaggcggcatacgccttgcaggaggcggttgacgcagtagcgaaggcagtggcagatgtg |
| CCTα-8pA-FL R | catctgccactgccttcgctactgcgtcaaccgcctcctgcaaggcgtatgccgcctcgttg |
| Lipin1α-mCherry F | gacaccggtcgccaccatggtgagcaagggcgaggag |
| Lipin1α-mCherry R | ataagaatgcggccgctttacttgtacagctcgtccatg |
| Lipin1α-∆M-Lip F | gagcacctcggacaaggaggaaagtaagccag |
| Lipin1α-∆M-Lip R | cttactttcctccttgtccgaggtgctctccac |
| NLS^c-myc^-Lipin1α-left F | accgtcagatccgctagc |
| NLS^c-myc^-Lipin1α-left R | aacttgaccctcttggcagcaggtactacagagctgcttg |
| NLS^c-myc^-Lipin1α-right F | ctgccaagagggtcaagttggactcacagctggacagcctg |
| NLS^c-myc^-Lipin1α-right R | gaagcttgagctcgag |
| Lipin1α-∆M-Lip^CT^-left R | ctggcttactttcctccttgggcattttatc |
| Lipin1α-∆M-Lip^CT^-right F | aaggaggaaagtaagccag |
| NLS^c-myc^-EGFP/mCherry F | gatccgctagcgctaccggtcgccaccatgcctgctgccaagagggtcaagttggacgtgagcaagggcgaggag |
| NLS^c-myc^-EGFP/mCherry R: | tccggacttgtacagctcgtc |
| NLS^c-myc^-M-Lip F | cgagctgtacaagtccggagggctgagggacctc |
| NLS^c-myc^-M-Lip R | gactgcagaattcgaagcttgttagattgtggtgtttcttc |
| NLS^c-myc^-EGFP-empty R | gactgcagaattcgaagcttg |
| HisGFPormCherry F | gtattttcagggcgccatgggatccgtgagcaagggcgaggag |
| HisLact^C2^ R | gattactttctgttcgacttaagctaacagcccagcagctccac |
| HisNLSmCherryLact^C2^ F | gtattttcagggcgccatgggatcctcttctggtcctattcgaac |
| HisERLmCherryLact^C2^ F | gtattttcagggcgccatgggatccgtgaagctctccctggtggccgcgatg |
| HisERLmCherryLact^C2^ R | gattactttctgttcgacttaagcta**tagctcgtcttt**acagcccagcagctccactc |
| HismCherryEvt^2xPH^ R | gattactttctgttcgacttaaggatccggtggatccctagtttgtc |
| HisEGFPCCTα(1-255) R | gattactttctgttcgacttaagttaatctttcactttcttctttac |

^a^: Black bold letters indicate the sequences of oligonucleotides encoding NLS/2NES/ER signal peptide/ ER retrieval sequence KDEL/ CAAX sequence “CLLL”.
