# Supplementary material for: Phosphatidylserine enrichment in the nuclear membrane regulates key enzymes of phosphatidylcholine synthesis
Source: EMBO J. 2024 Jun 25;43(16):3414–49. doi: 10.1038/s44318-024-00151-z (PMC11329639; doi:10.1038/s44318-024-00151-z)
Supplement: Supplementary file 2 — Table EV2 [file 44318_2024_151_MOESM2_ESM.doc]

**Table EV2: Reagents and Tools:**

| Reagent or Resource | Source | Identifier |
| --- | --- | --- |
| Antibodies |  |  |
| Mouse anti c-MYC | Invitrogen | Cat# 13-2500 |
| Chemicals, Peptides, and Recombinant Proteins |  |  |
| Oleic Acid | Sigma-aldrich | Cat# 01008 |
| HCS LipidTOX^TM^ Deep Red | Invitrogen | Cat# H34477 |
| Lipofectamine 2000 | Invitrogen | Cat# 11668-019 |
| 16% Paraformaldehyde Solution | Electron Microscopy Sciences | Cat# 15710 |
| Cell lines |  |  |
| U2OS | ATCC | HTB-96 |
| HeLa | ATCC | CCL-2 |
| Huh7 | AcceGen | ABC-TC0437 |
| Oligonucleotides |  |  |
| See Table S1 | Integrated DNA Technologies IDT | N/A |
| Recombinant DNA |  |  |
| NLS-mCherry-Lact^C2^ | This paper | N/A |
| NLS-mCherry-Lact^C2,AAA^ | This paper | N/A |
| NLS-mCherry-Evt^2xPH^ | This paper | N/A |
| NLS-mCherry-Evt^2xPH,K20E^ | This paper | N/A |
| ERLum-mCherry-Lact^C2^ | This paper | N/A |
| ERLum-mCherry-Evt^2xPH^ | This paper | N/A |
| pCDNA3-myc-yPSD1 WT | A gift form Professor Tomohiko Taguchi | N/A |
| pCDNA3-myc-yPSD1 S463A | A gift form Professor Tomohiko Taguchi | N/A |
| NLS-myc-yPSD1-IRES2-EGFP | This paper | N/A |
| NLS-myc-yPSD1^S463A^-IRES2-EGFP | This paper | N/A |
| 2NES-myc-yPSD1-IRES2-EGFP | This paper | N/A |
| 2NES-myc-yPSD1^S463A^-IRES2-EGFP | This paper | N/A |
| NLS-myc-yPSD1-IRES2-EGFP (wo IRES2-EGFP) | This paper | N/A |
| NLS-myc-yPSD1^S463A^-IRES2-EGFP (wo IRES2-EGFP) | This paper | N/A |
| 2NES-myc-yPSD1-IRES2-EGFP (wo IRES2-EGFP) | This paper | N/A |
| 2NES-myc-yPSD1^S463A^-IRES2-EGFP (wo IRES2-EGFP) | This paper | N/A |
| EGFP-Emerin | A gift from Eric Schirmer | Addgene plasmid #61993 |
| mCherry-Emerin | This paper | N/A |
| HaloTag-Emerin | This paper | N/A |
| PSS1-HaloTag | This paper | N/A |
| PSS1^Q353R^-HaloTag | This paper | N/A |
| mCherry-Sec61β | A gift from Jennifer Lippincott-Schwartz | N/A |
| HaloTag-Sec61β | A gift from Jennifer Lippincott-Schwartz | N/A |
| mEmerald-KDEL | A gift from Jennifer Lippincott-Schwartz | N/A |
| CCTα-EGFP | This paper | N/A |
| CCTα-mCherry | This paper | N/A |
| pET-28b(+)-LIPIN1α | A gift from Dr. George Carman | N/A |
| LIPIN1α-EGFP | This paper | N/A |
| LIPIN1α-mCherry | This paper | N/A |
| CCTα-8pQ-FL-EGFP | This paper | N/A |
| CCTα-8pA-FL-EGFP | This paper | N/A |
| EGFP-CCTα(1-225) | This paper | N/A |
| EGFP-CCTα(1-225)-CAAX | This paper | N/A |
| EGFP-CCTα(1-225)-4hQ-CAAX | This paper | N/A |
| EGFP-CCTα(1-225)-8pQ-CAAX | This paper | N/A |
| EGFP-CCTα(1-225)-12Q-CAAX | This paper | N/A |
| NLS(c-myc)-Lipin1α-EGFP | This paper | N/A |
| Lipin1α-∆M-Lip-EGFP | This paper | N/A |
| Lipin1α-∆M-Lip^CT^-EGFP | This paper | N/A |
| NLS(c-myc)-mCherry-M-Lip | This paper | N/A |
| NLS(c-myc)-EGFP-M-Lip | This paper | N/A |
| NLS(c-myc)-EGFP-Vector | This paper | N/A |
| His-tagged-EGFP-Lact^C2^ | This paper | N/A |
| His-tagged-EGFP-Lact^C2,AAA^ | This paper | N/A |
| His-tagged-mCherry-Lact^C2^ | This paper | N/A |
| His-tagged-NLS-mCherry-Lact^C2^ | This paper | N/A |
| His-tagged-ER^Lum^-mCherry-Lact^C2^-KDEL | This paper | N/A |
| His-tagged-mCherry-Evt^2xPH^ | This paper | N/A |
| His-tagged-EGFP-CCTa(1-255) | This paper | N/A |
| software |  |  |
| GraphPad Prism 6 | GraphPad Prism software | https://www.graphpad.com/support/prism-6-updates/ |
| Fiji | Fiji | https://imagej.net/software/fiji/downloads |
| Adobe Illustrator Adobe | Adobe | https://www.adobe.com/ |
| Shotcut | Shotcut | https://shotcut.org/ |
