# Supplementary material for: Phosphatidylserine enrichment in the nuclear membrane regulates key enzymes of phosphatidylcholine synthesis
Source: EMBO J. 2024 Jun 25;43(16):3414–49. doi: 10.1038/s44318-024-00151-z (PMC11329639; doi:10.1038/s44318-024-00151-z)
Supplement: Supplementary file 3 — Appendix [file 44318_2024_151_MOESM3_ESM.pdf]

Appendix

PHOSPHATIDYLSERINE ENRICHMENT IN THE NUCLEAR MEMBRANE REGULATES KEY ENZYMES OF PHOSPHATIDYLCHOLINE SYNTHESIS

Yang Niu[1, #], Joshua G. Pemberton[1], Yeun Ju Kim[1] and Tamas Balla[1, #]

[1]Section on Molecular Signal Transduction, *Eunice Kennedy Shriver* National Institute of Child Health and Human Development, National Institutes of Health, Bethesda, MD 20892

[#] Corresponding Authors:

E-mail: niuyang001@outlook.com and ballat@mail.nih.gov

Appendix Figure S1 Page 2

Appendix Figure S2 Page 3

Appendix Figure S3 Page 4

Appendix Figure S4 Page 5

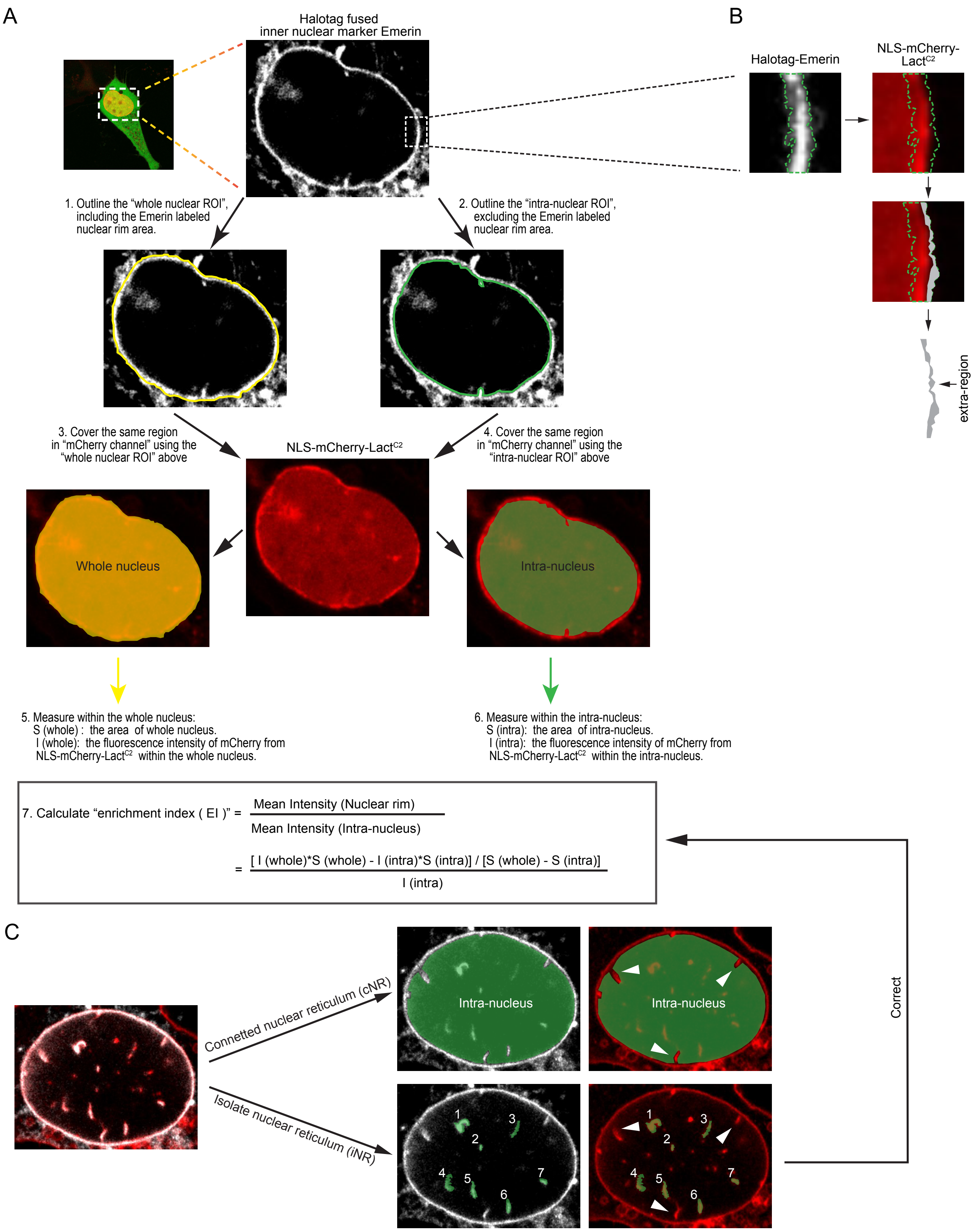

Workflow for calculation of membrane enrichment of NLS-mCherry-LactC2 to the INM and NR.
(A-C) Schematics of the various steps used for calculation of the enrichment index (EI) of a particular lipid probe in the INM (related to the image analysis described in Methods).

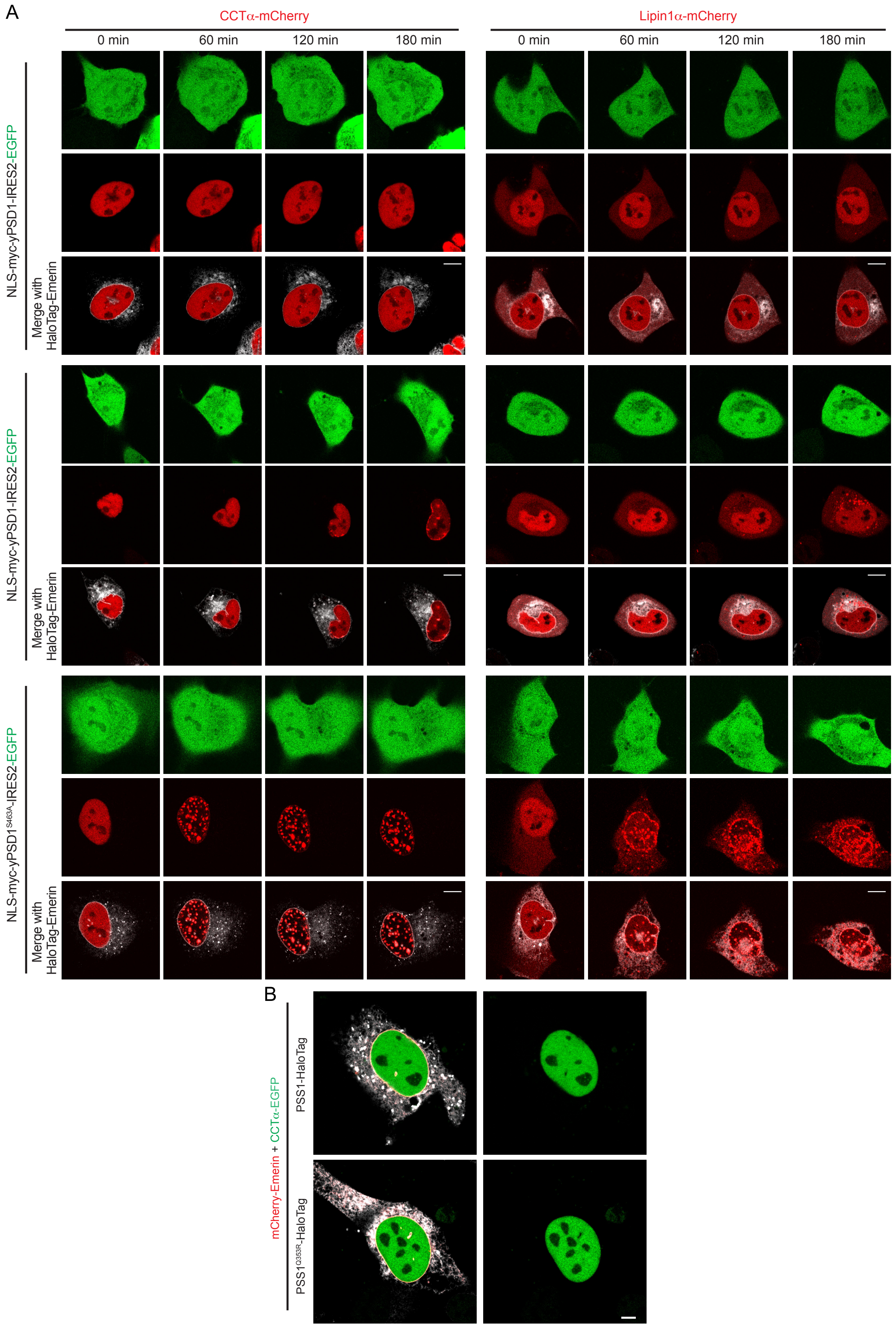

PS plays a role in the INM translocation of CCTα and Lipin1α in response to oleic acid (OA) treatment. (A) Representative images of U2OS cells expressing CCTα-mCherry (left) or Lipin1α- mCherry (right) together with HaloTag-Emerin (gray) and NLS-myc-yPSD1-IRES2-EGFP or the yPSD1^{S463A} mutant version. Time-lapse images show the effects of treatment with OA for the indicated times. Top panels show complete lack of translocation of the enzymes to the NM and NR in cells expressing high level of the yPSD1 construct, while middle panels show a partial response in cells that show less yPSD1 enzyme. Note the massive translocation of the CCTα and Lipin1α enzymes in the cells expressing the inactive yPSD1 enzyme shown in the lower panels. Scale bar, 10 µm. (see also Supplementary Videos 18 and 19). (B) Representative images of U2OS cells expressing CCTα-mCherry together with mCherry-Emerin and HaloTag PSS1 WT- or the PSS1^{Q353R} mutant without OA treatment. Note that even high production of PS by the PSS1 enzymes fails to cause translocation of the CCTα protein to INM or NR. Scale bar, 10 µm.

Appendix Fig. S2

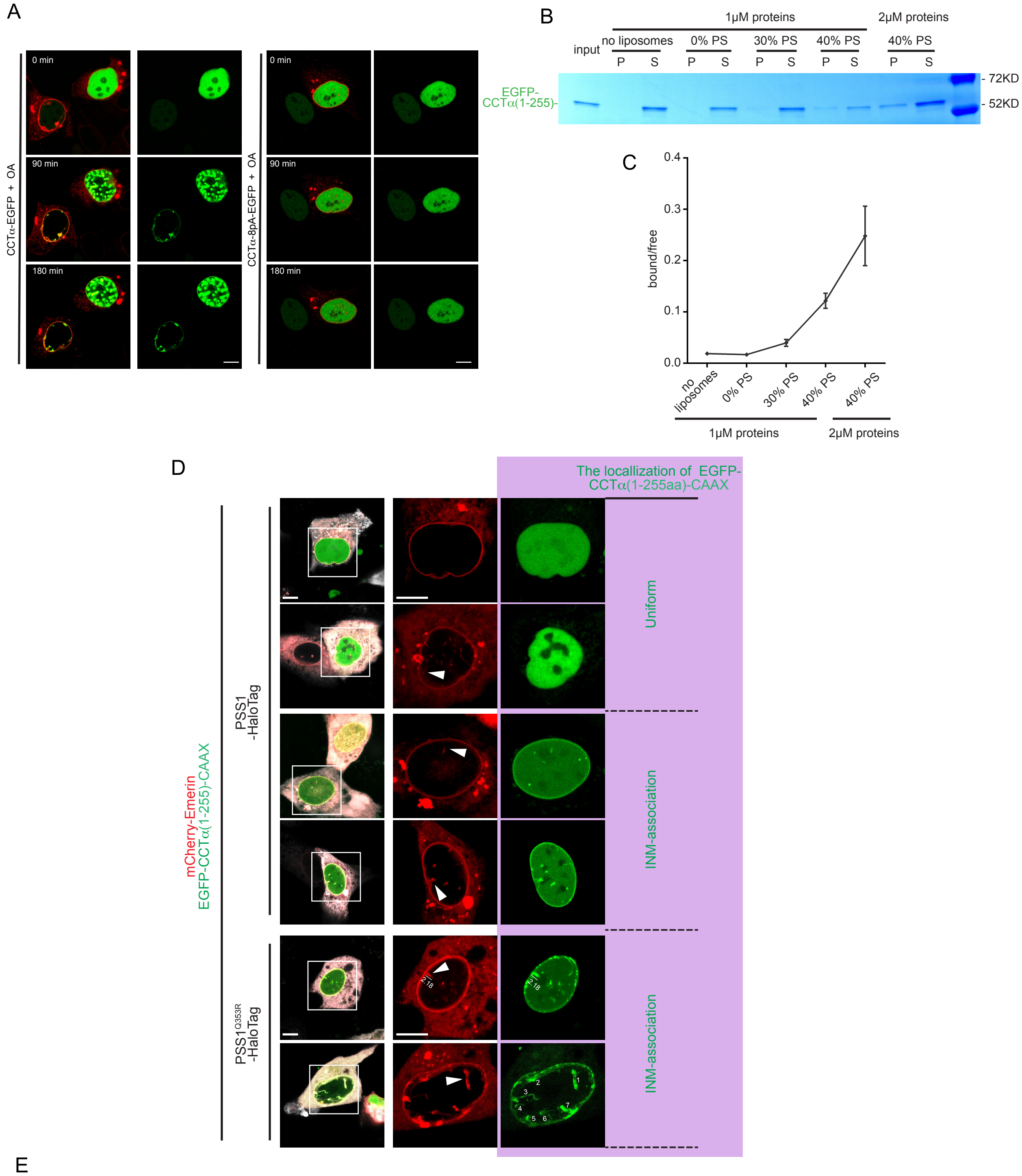

E

	PSS1-HaloTag (uniform CCTα(1-255)-CAAX)	PSS1-HaloTag (INM-associated CCTα(1-255)-CAAX)	PSS1^{Q353R}-HaloTag (INM-associated CCTα(1-255)-CAAX)
NRs lableled by mCherry-Emerin:			
−: no NRs observed	− (297 cells in total 344 cells)	− (116 cells in total 217 cells)	− (112 cells in total 357 cells)
+: lengh of all NRs < 2μm	+ (47 cells in total 344 cells)	+ (78 cells in total 217 cells)	+ (125 cells in total 357 cells)
++: 3 ≥ Number (length ≥ 2μm) of NRs >0		++ (23 cells in total 217 cells)	++ (67 cells in total 357 cells)
+++: Number (length ≥ 2μm) of NRs > 3			+++ (53 cells in total 357 cells)

PS binding is critical for the OA-induced INM association of CCTα mediated by its M domain.
(A) Selected images from a time-lapse showing U2OS cells transiently expressing mCherry-Emerin and either the full length wild-type CCTα (CCTα-WT-FL-EGFP) or a mutant form in which the positive residues within the N-terminal end of the M-domain were replaced by alanine residues (CCTα-8pA-EGFP) in their response to OA treatment. Scale bar, 10 μm.
(B,C) The result of a representative of three SDS-PAGE analyses showing protein-liposome co-sedimentation assays using recombinant EGFP-CCTα(1-255) and liposomes containing increasing amounts of PS. (c) Results of quantification from 3 experiments shown in (B). Data shown are means ± SEM. Note that binding can be observed but only at high PS concentrations and using more protein to liposome ratios in the assay. (D) Representative images of live U2OS cells transiently expressing EGFP-CCTα(1-255)-CAAX, mCherry-Emerin, together with PSS1 or the PSS1^{Q353R} mutant with a HaloTag. The enlarged nuclear regions from the left columns are showed in the right two columns. White arrowheads indicate typical NR structures labeled by mCherry-Emerin (red). The length of a typical NR a little longer than 2 μm was marked by its length, 2.18 μm. The white numbers in the bottom row mark NRs that are longer than 2 μm in one nucleus. Scale bar, 10 μm. Note that increased PS production by overexpression of wild-type PSS1 increases association of EGFP-CCTα(1-255)-CAAX with the INM and NR in some but not all cells, whereas stronger INM and NR association and expansion of NR is seen in cells expression the hyperactive PSS1^{Q353R} mutant.
(E) A Table summarizing the fraction of cells with different degrees of INM/NR localization.

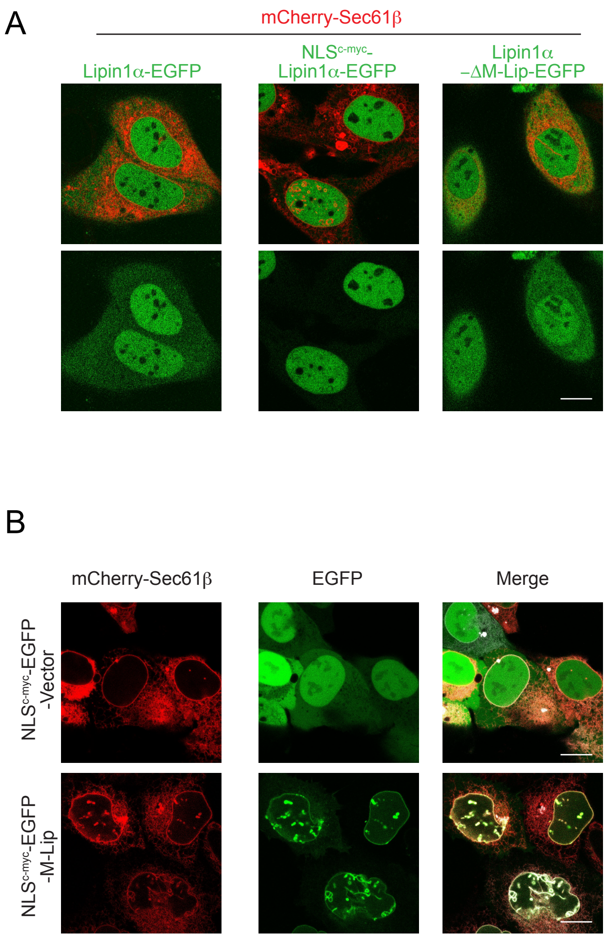

Nuclear localization of Lipin1α or its mutant forms and ability of the isolated M-Lip domain to associate with the NR and INM.

(A) Representative confocal images of live U2OS cells transiently expressing mCherry-Sec61β, together with EGFP-Lipin1α or mutant variants, showing their resting nuclear localization. Scale bar, 10 μm.

(B) Representative confocal images of live U2OS cells transiently expressing HaloTag-Emerin (gray) (not displayed), mCherry-Sec61β (red), together with EGFP-M-Lip targeted to the nucleus by NLS (from c-myc).

Note that this construct associates with the INM and NR even without OA treatment. Scale bar, 10 μm.

Appendix Fig. S4
