# Supplementary material for: Phosphatidylserine enrichment in the nuclear membrane regulates key enzymes of phosphatidylcholine synthesis
Source: EMBO J. 2024 Jun 25;43(16):3414–49. doi: 10.1038/s44318-024-00151-z (PMC11329639; doi:10.1038/s44318-024-00151-z)
Supplement: Supplementary file 5 — Movie EV1 [file 44318_2024_151_MOESM5_ESM.zip › Readme to Movie EV1.docx]

**Movie EV1. Nuclear targeted PS biosensor (NLS-mCherry-Lact^C2^) reveals the enrichment of PS in INM.** Z-stacks generated from live-cell imaging of U2OS cell transiently expressing NLS-mCherry-Lact^C2^ (red) and nuclear membrane marker EGFP-Emerin (green). Scale bar, 10 µm.
