# Supplementary material for: Phosphatidylserine enrichment in the nuclear membrane regulates key enzymes of phosphatidylcholine synthesis
Source: EMBO J. 2024 Jun 25;43(16):3414–49. doi: 10.1038/s44318-024-00151-z (PMC11329639; doi:10.1038/s44318-024-00151-z)
Supplement: Supplementary file 6 — Movie EV2 [file 44318_2024_151_MOESM6_ESM.zip › Readme to Movie EV2.docx]

**Movie EV2. Uniform distribution of ER^Lum^-mCherry-Lact^C2^ within the ER lumen, designated as Pattern 1.** Time lapse images of U2OS cell transiently expressing ER^Lum^-mCherry-Lact^C2^ (red) and ER marker mEmerald-Sec61β under hypotonic condition. White arrows indicate the gradual separation of the membrane of the swelling ER. Scale bar, 1 μm.
