# Supplementary material for: Phosphatidylserine enrichment in the nuclear membrane regulates key enzymes of phosphatidylcholine synthesis
Source: EMBO J. 2024 Jun 25;43(16):3414–49. doi: 10.1038/s44318-024-00151-z (PMC11329639; doi:10.1038/s44318-024-00151-z)
Supplement: Supplementary file 7 — Movie EV3 [file 44318_2024_151_MOESM7_ESM.zip › Readme to Movie EV3.docx]

**Movie EV3. Subtle enrichment of ER^Lum^-mCherry-Lact^C2^ in the luminal leaflet of the ER membrane (LER), designated as Pattern 2.** Time-lapse images of U2OS cell transiently expressing ERLum-mCherry-Lact^C2^ (red) and the ER marker mEmerald-Sec61β under hypotonic condition. White arrows indicate the gradual separation of the membrane of the swelling ER. Yellow arrowheads indicate the subtle localization of Lact^C2^ in the LER. Scale bar, 1 μm.
