# Supplementary material for: Phosphatidylserine enrichment in the nuclear membrane regulates key enzymes of phosphatidylcholine synthesis
Source: EMBO J. 2024 Jun 25;43(16):3414–49. doi: 10.1038/s44318-024-00151-z (PMC11329639; doi:10.1038/s44318-024-00151-z)
Supplement: Supplementary file 8 — Movie EV4 [file 44318_2024_151_MOESM8_ESM.zip › Readme to Movie EV4.docx]

**Movie EV4. Moderate enrichment of ER^Lum^-mCherry-Lact^C2^ in the luminal leaflet of the ER membrane (LER), designated as Pattern 3.** Time-lapse images of U2OS cell transiently expressing ERLum-mCherry-Lact^C2^ (red) and the ER marker mEmerald-Sec61β under hypotonic condition. Scale bar, 1 μm.
