# Supplementary material for: Phosphatidylserine enrichment in the nuclear membrane regulates key enzymes of phosphatidylcholine synthesis
Source: EMBO J. 2024 Jun 25;43(16):3414–49. doi: 10.1038/s44318-024-00151-z (PMC11329639; doi:10.1038/s44318-024-00151-z)
Supplement: Supplementary file 11 — Movie EV7 [file 44318_2024_151_MOESM11_ESM.zip › Readme to Movie EV7.docx]

**Movie EV7. Enrichment of ER^Lum^-mCherry-Lact^C2^ in the luminal leaflet of the ER membrane (LER) in U2OS cells expressing PSS1^Q353R^-HaloTag.** Time lapse images of U2OS cell transiently expressing PSS1^Q353R^-HaloTag (gray), ER^Lum^-mCherry-Lact^C2^ (red) and the soluble ER-luminal marker mEmerald-KDEL as they respond to hypotonic conditions. White arrows indicate the gradual separation of the ER membranes during swelling. Scale bar, 1 μm.
