# Supplementary material for: Phosphatidylserine enrichment in the nuclear membrane regulates key enzymes of phosphatidylcholine synthesis
Source: EMBO J. 2024 Jun 25;43(16):3414–49. doi: 10.1038/s44318-024-00151-z (PMC11329639; doi:10.1038/s44318-024-00151-z)
Supplement: Supplementary file 14 — Movie EV10 [file 44318_2024_151_MOESM14_ESM.zip › Readme to Movie EV10.docx]

**Movie EV10. Nuclear blebs in U2OS cells generated by hypotonic challenge show that NLS-mCherry-Lact^C2^ is confined to the INM, but not ONM, both in the absence and presence of PSS1 expression.** Time-lapse images of live U2OS cells expressing NLS-mCherry-Lact^C2^ (red) and the ER marker mEmerald-Sec61β (green) are shown first, followed by the enlarged images of nuclear blebs from the ROIs marked by white boxes. In the subsequent enlarged images (without PSS1-HaloTag expression), NLS-mCherry-Lact^C2^ (red), the ER marker mEmerald-Sec61β (green), and the merged channels are shown. Note, especially in case #1, which also shows the strong plasma membrane signal from the fraction of the PS probe leaked into the cytoplasmic side, that the signal is only detected in the nuclear leaflet of the INM. The subsequent images show cells that also express PSS1 WT-HaloTag (white), together with NLS-mCherry-Lact^C2^ (red) and, in this case, the luminal soluble ER marker mEmerald-KDEL (green). White and yellow arrows, respectively, indicate the ONM and INM of the gradual separation of nuclear envelope membrane during swelling. Note that only the INM is decorated by the PS sensor Lact^C2^ from the nucleoplasm. Scale bar, 1 μm.
