# Supplementary material for: Phosphatidylserine enrichment in the nuclear membrane regulates key enzymes of phosphatidylcholine synthesis
Source: EMBO J. 2024 Jun 25;43(16):3414–49. doi: 10.1038/s44318-024-00151-z (PMC11329639; doi:10.1038/s44318-024-00151-z)
Supplement: Supplementary file 15 — Movie EV11 [file 44318_2024_151_MOESM15_ESM.zip › Readme to Movie EV11.docx]

**Movie EV11. Lack of membrane localization of the mCherry-Lact^C2^ probe expressed in the cytoplasm to the nuclear membrane or the ER membrane facing the cytoplasm in U2OS cells, even in the presence of PSS1 WT expression.** In the first movie sequence, cells expressing the ER marker mEmerald-Sec61β (green) and the cytoplasmic mCherry-Lact^C2^ (red) are shown together with the merged channels as they respond to a hypoosmotic challenge. In the second sequence, cells expressing PSS1 (grey) together with the luminal soluble ER marker, mEmerald-KDEL (green) and the cytoplasmic mCherry-Lact^C2^ (red) and their response to hypotonic conditions is shown. White arrows indicate the gradual separation of the ER membranes during swelling. Note the lack of Lact^C2^ signal on ER structures when accessed from the cytoplasm. Scale bar, 1 μm.
