# Supplementary material for: Phosphatidylserine enrichment in the nuclear membrane regulates key enzymes of phosphatidylcholine synthesis
Source: EMBO J. 2024 Jun 25;43(16):3414–49. doi: 10.1038/s44318-024-00151-z (PMC11329639; doi:10.1038/s44318-024-00151-z)
Supplement: Supplementary file 16 — Movie EV12 [file 44318_2024_151_MOESM16_ESM.zip › Readme to Movie EV12.docx]

**Movie EV12. Recruitment of CCTα from the nucleoplasm to the NR and INM upon oleic acid (OA) treatment.** U2OS cells transiently expressing CCTα-EGFP (green), mCherry-Emerin (red) and HaloTag-Sec61β (gray) were exposed to OA treatment. Scale bar, 5 µm.
