# Supplementary material for: Phosphatidylserine enrichment in the nuclear membrane regulates key enzymes of phosphatidylcholine synthesis
Source: EMBO J. 2024 Jun 25;43(16):3414–49. doi: 10.1038/s44318-024-00151-z (PMC11329639; doi:10.1038/s44318-024-00151-z)
Supplement: Supplementary file 18 — Movie EV14 [file 44318_2024_151_MOESM18_ESM.zip › Readme to Movie EV14.docx]

**Movie EV14. Nuclear membrane dynamics of U2OS cells transiently expressing EGFP-Vector, mCherry-Emerin and HaloTag-Sec61β in response to OA treatment.** EGFP-Vector (green), mCherry-Emerin (red), and HaloTag-Sec61β (gray). Scale bar, 5 µm.
