# Supplementary material for: Phosphatidylserine enrichment in the nuclear membrane regulates key enzymes of phosphatidylcholine synthesis
Source: EMBO J. 2024 Jun 25;43(16):3414–49. doi: 10.1038/s44318-024-00151-z (PMC11329639; doi:10.1038/s44318-024-00151-z)
Supplement: Supplementary file 19 — Movie EV15 [file 44318_2024_151_MOESM19_ESM.zip › Readme to Movie EV15.docx]

**Movie EV15. Parallel recruitment of CCTα and Lipin1α from the nucleoplasm to the NR and INM in U2OS cells transiently expressing CCTα-mCherry, Lipin1α-EGFP, and HaloTag-Emerin in response to OA treatment.** Lipin1α-EGFP (green), CCTα-mCherry (red), HaloTag-Emerin (gray). Scale bar, 5 µm.
