# Supplementary material for: Phosphatidylserine enrichment in the nuclear membrane regulates key enzymes of phosphatidylcholine synthesis
Source: EMBO J. 2024 Jun 25;43(16):3414–49. doi: 10.1038/s44318-024-00151-z (PMC11329639; doi:10.1038/s44318-024-00151-z)
Supplement: Supplementary file 21 — Movie EV17 [file 44318_2024_151_MOESM21_ESM.zip › Readme to Movie EV17.docx]

**Movie EV17. The localization between Lipin1α and NLS-mCherry-Lact^C2^ (at low expression level) in U2OS cells in response to OA treatment.** Lipin1α-EGFP (green), NLS-mCherry-Lact^C2^ (red), HaloTag-Emerin (grey), and Hoechst (blue). Scale bar, 10 µm.
