# Supplementary material for: Phosphatidylserine enrichment in the nuclear membrane regulates key enzymes of phosphatidylcholine synthesis
Source: EMBO J. 2024 Jun 25;43(16):3414–49. doi: 10.1038/s44318-024-00151-z (PMC11329639; doi:10.1038/s44318-024-00151-z)
Supplement: Supplementary file 22 — Movie EV18 [file 44318_2024_151_MOESM22_ESM.zip › Readme to Movie EV18.docx]

**Movie EV18. The oleic acid (OA)-induced recruitment of CCTα from the nucleoplasm to the NR and INM is impaired by nuclear expression of a yeast phosphatidylserine decarboxylase (yPSD1).** U2OS cells transiently expressed CCTα-mCherry (red), HaloTag-Emerin (grey) and either NLS-myc-yPSD1 or S463A mutant with an IRES2-EGFP (green). Their response to OA treatment was followed in a time-lapse. Note that EGFP is not fused to the yPSD1 but expressed separately from the same plasmid only to mark the cells expressing the myc-tagged yPSD1 enzyme. Expression of the WT but not the inactive yPSD1^S463A^ mutant (top row) greatly reduced (middle row) or completely eliminated (bottom row) the recruitment of CCTα to the INM and NR. Scale bar, 10 µm.
