# Supplementary material for: Phosphatidylserine enrichment in the nuclear membrane regulates key enzymes of phosphatidylcholine synthesis
Source: EMBO J. 2024 Jun 25;43(16):3414–49. doi: 10.1038/s44318-024-00151-z (PMC11329639; doi:10.1038/s44318-024-00151-z)
Supplement: Supplementary file 23 — Movie EV19 [file 44318_2024_151_MOESM23_ESM.zip › Readme to Movie EV19.docx]

**Movie EV19. The oleic acid (OA)-induced recruitment of Lipin1α from the nucleoplasm to the NR and INM is impaired by nuclear expression of a yeast phosphatidylserine decarboxylase (yPSD1).** U2OS cells transiently expressed Lipin1α-mCherry (red), HaloTag-Emerin (gray) and either NLS-myc-yPSD1 WT or S463A mutant with and IRES2-EGFP (green). Their response to OA treatment was followed in a time lapse. Note that GFP is not fused to the yPSD but expressed separately from the same plasmid only to mark the cells expressing the myc-tagged yPSD1 enzyme. Note that expression of WT but not the inactive yPSD1^S463A^ mutant (top row) greatly reduced (middle row) or completely eliminated (bottom row) the recruitment of Lipin1α to the INM and NR. Scale bar, 10 µm.
