# Supplementary material for: Phosphatidylserine enrichment in the nuclear membrane regulates key enzymes of phosphatidylcholine synthesis
Source: EMBO J. 2024 Jun 25;43(16):3414–49. doi: 10.1038/s44318-024-00151-z (PMC11329639; doi:10.1038/s44318-024-00151-z)
Supplement: Supplementary file 24 — Movie EV20 [file 44318_2024_151_MOESM24_ESM.zip › Readme to Movie EV20.docx]

**Movie EV20. Time lapse images of nuclear membrane dynamics in U2OS cells transiently expressing NLS^c-myc^-EGFP-M-Lip domain in response to OA treatment.** HaloTag-Sec61β (gray), mCherry-Emerin (red) and NLS^c-myc^-EGFP-M-Lip (green)**.** Note the constitutive localization of the M-Lip domain to the NM and NR, which does not change after OA exposure. Scale bar, 10 µm.
