# Supplementary material for: Phosphatidylserine enrichment in the nuclear membrane regulates key enzymes of phosphatidylcholine synthesis
Source: EMBO J. 2024 Jun 25;43(16):3414–49. doi: 10.1038/s44318-024-00151-z (PMC11329639; doi:10.1038/s44318-024-00151-z)
Supplement: Supplementary file 30 — Expanded View Figures [file 44318_2024_151_MOESM30_ESM.pdf]

Expanded View Figures

Figure EV1. Monitoring PS levels in the inner nuclear membrane (INM) using engineered biosensors.

Related to Fig. 1. (**A**, **B**) A representative result of one of four SDS-PAGE analyses of protein–liposome co-sedimentation assay comparing the PS binding of recombinant mCherry-Lact^{C2} constructs fused to various targeting sequences. This analysis did not reveal any difference among the three (cytoplasm/ER lumen/nucleus-targeted) Lact^{C2} variants in their PS affinities. Data shown are mean ± SEM ($n = 4$ independent experiments). (**C**, **D**) A representative result of one of four SDS-PAGE analyses of protein–liposome co-sedimentation assay comparing the PS-binding affinities of recombinant mCherry-Lact^{C2} and mCherry-Evt^{2xPH} domains. This analysis showed a weaker affinity of Evt^{2xPH} than Lact^{C2} toward PS-containing liposomes. Data shown are mean ± SEM ($n = 4$ independent experiments). (**E**) Live-cell confocal images of U2OS cells expressing EGFP-Emerin and either mCherry-Lact^{C2} or mCherry-Lact^{C2,AAA} targeted to the nucleus. The areas within the nucleus (marked by the dotted circles) were subjected to repeated photobleaching to reduce the fluorescent signal. Such repeated photobleaching did not affect the nuclear membrane attachment of NLS-mCherry-Lact^{C2} (upper panel, representative of 20 cells from 3 independent experiments), but clearly showed no membrane-associated signal in the cells with the high nuclear accumulation of the NLS-mCherry-Lact^{C2,AAA} construct (lower panel, 20 cells, $n = 3$ independent experiments). Scale bar, 10 µm. (**F**) Similar photobleaching regime as shown in (**E**) using U2OS cells overexpressing EGFP-Emerin, PSS1^{Q353R}-HaloTag and either NLS-mCherry-Evt^{2xPH} (upper panels, 20 cells, $n = 3$ independent experiments) or NLS-mCherry-Evt^{2xPH,K20E} (lower panel, 20 cells, $n = 3$ independent experiments). Note that even though the mutant Evt^{2xPH} probe shows a much higher nuclear accumulation, it does not bind to the nuclear membrane. Scale bar, 10 µm. (**G**) Confocal images of nuclei from the live-cell imaging of HeLa (left) or Huh7 (right) cells expressing the NLS-mCherry-Lact^{C2}. Scale bar, 10 µm.

This is a U.S. Government work and not under copyright protection in the US; foreign copyright protection may apply

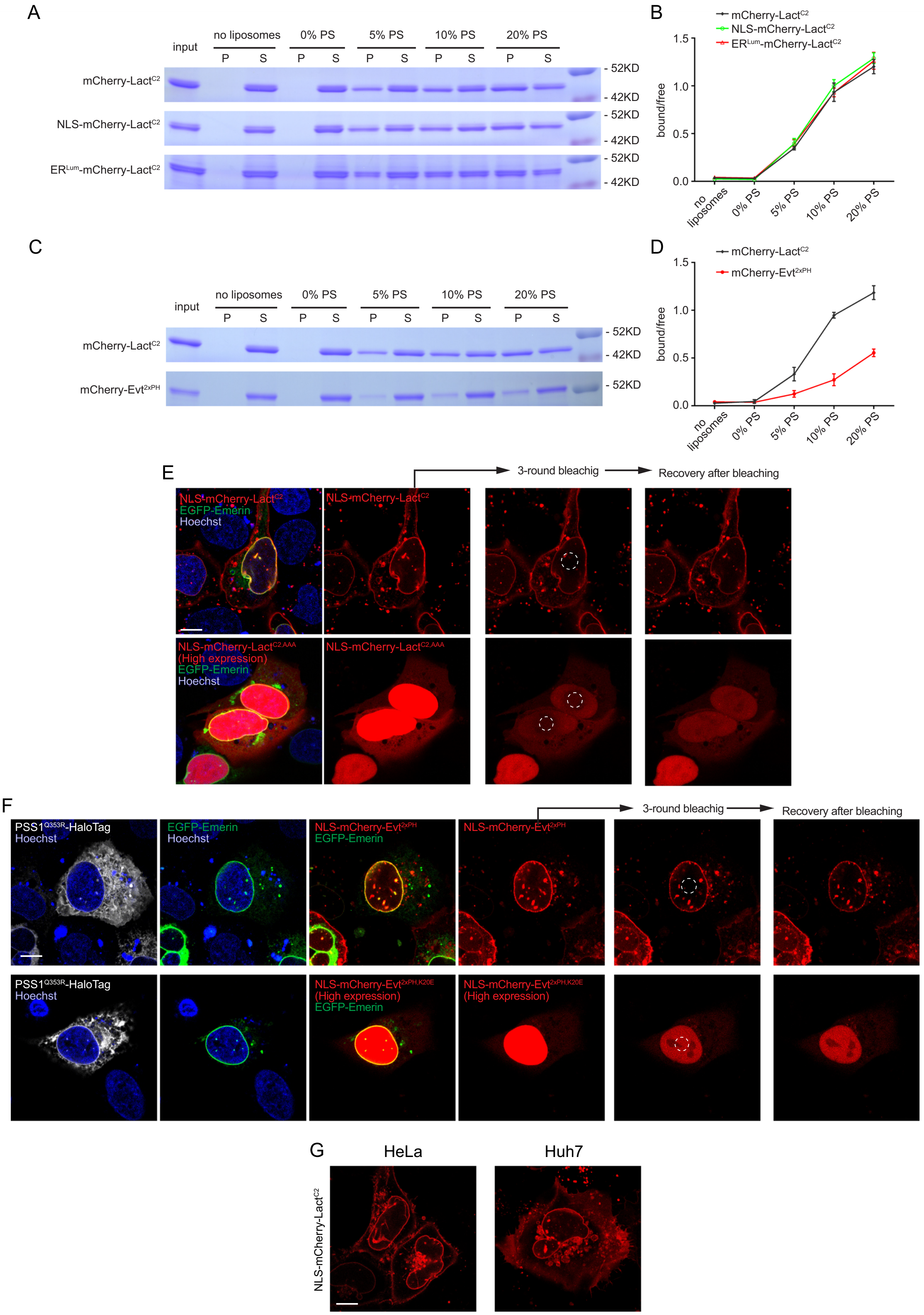

 This is a U.S. Government work and not under copyright protection in the US; foreign copyright protection may apply

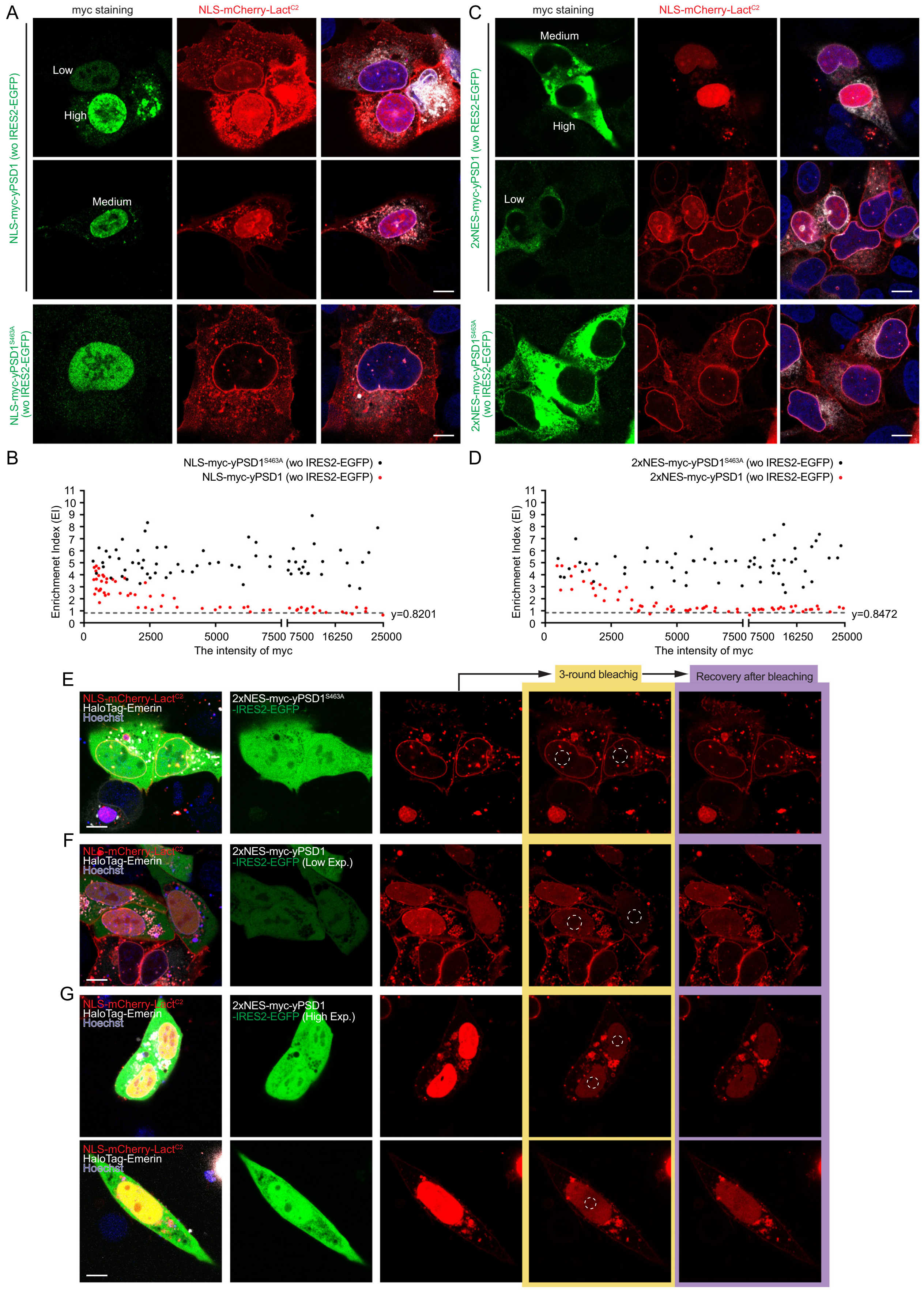
A
myc staining
NLS-mCherry-Lact C2
NLS-myc-yPSD1 (wo IRES2-EGFP)
Low
High
Medium
NLS-myc-yPSD1 S463A (wo IRES2-EGFP)
C
myc staining
NLS-mCherry-Lact C2
2xNES-myc-yPSD1 (wo RES2-EGFP)
Medium
High
Low
2xNES-myc-yPSD1 S463A (wo IRES2-EGFP)
B
NLS-myc-yPSD1 S463A (wo IRES2-EGFP)
NLS-myc-yPSD1 (wo IRES2-EGFP)
Enrichmenet Index (EI)
y=0.8201
The intensity of myc
D
2xNES-myc-yPSD1 S463A (wo IRES2-EGFP)
2xNES-myc-yPSD1 (wo IRES2-EGFP)
Enrichmenet Index (EI)
y=0.8472
The intensity of myc
E
3-round bleachig
Recovery after bleaching
NLS-mCherry-Lact C2
HaloTag-Emerin
Hoechst
2xNES-myc-yPSD1 S463A -IRES2-EGFP
F
2xNES-myc-yPSD1 -IRES2-EGFP (Low Exp.)
G
2xNES-myc-yPSD1 -IRES2-EGFP (High Exp.)

Figure EV2. Manipulation of nuclear PS levels by targeted PSD enzymes.

Related to Fig. 3. (**A**) U2OS cells transiently expressing NLS-mCherry-Lact^{C2} (red), HaloTag-fused Emerin (gray), and the NLS-myc-yPSD1 WT, or S463A mutant, this time without (wo) IRES2-EGFP. Cells were fixed and immuno-stained with anti-c-myc antibody. Representative images of cells showing various expression levels of NLS-myc-yPSD1. Scale bar, 10 μm. (**B**) The enrichment index (EI) of Lact^{C2} nuclear membrane localization as a function of NLS-myc-yPSD1 expression level based on myc staining intensity. Red dots represent cells expressing WT, while black dots show cells expressing the mutant NLS-myc-yPSD1^{S463A} (63 cells for NLS-myc-yPSD1 WT and 67 cells for NLS-myc-yPSD1-S463A, were scored from 3 independent experiments). The EI value of 0.8201, indicated by the horizontal dashed line, is the average EI value calculated from 7 cells that showed no INM localization of Lact^{C2}. (**C**, **D**) same as (**A**, **B**) using the 2xNES tagged myc-yPSD1 or S463A mutant (65 cells for 2xNES-myc-yPSD1 and 61 cells for 2xNES-myc-yPSD1^{S463A} were scored from 3 independent experiments). The EI value of 0.8472, indicated by the horizontal dashed line, is the average EI value calculated from 6 cells showing no INM localization of Lact^{C2}. Scale bar, 10 μm. (**E–G**) Assessing membrane localization of NLS-mCherry-Lact^{C2} in cells with high signal in the nucleus. Cells that express high level of 2xNES-myc-yPSD1-IRES2-EGFP show high amounts of NLS-mCherry-Lact^{C2} in the nucleus (**G**). When such cells are subjected to repeated photobleaching of a small area within the nucleus (labeled with dotted circles) to reduce the fluorescent signal, still no sign of membrane localization is observed. Note that membrane localization of NLS-mCherry-Lact^{C2} was still visible after a similar photobleaching regime in cells that express lower level of 2xNES-myc-yPSD1-IRES2-EGFP (**F**) or the inactive yPSD1^{S463A} (**E**) (20 cells scored for each of these groups from 3 experiments). Scale bar, 10 μm.

This is a U.S. Government work and not under copyright protection in the US; foreign copyright protection may apply

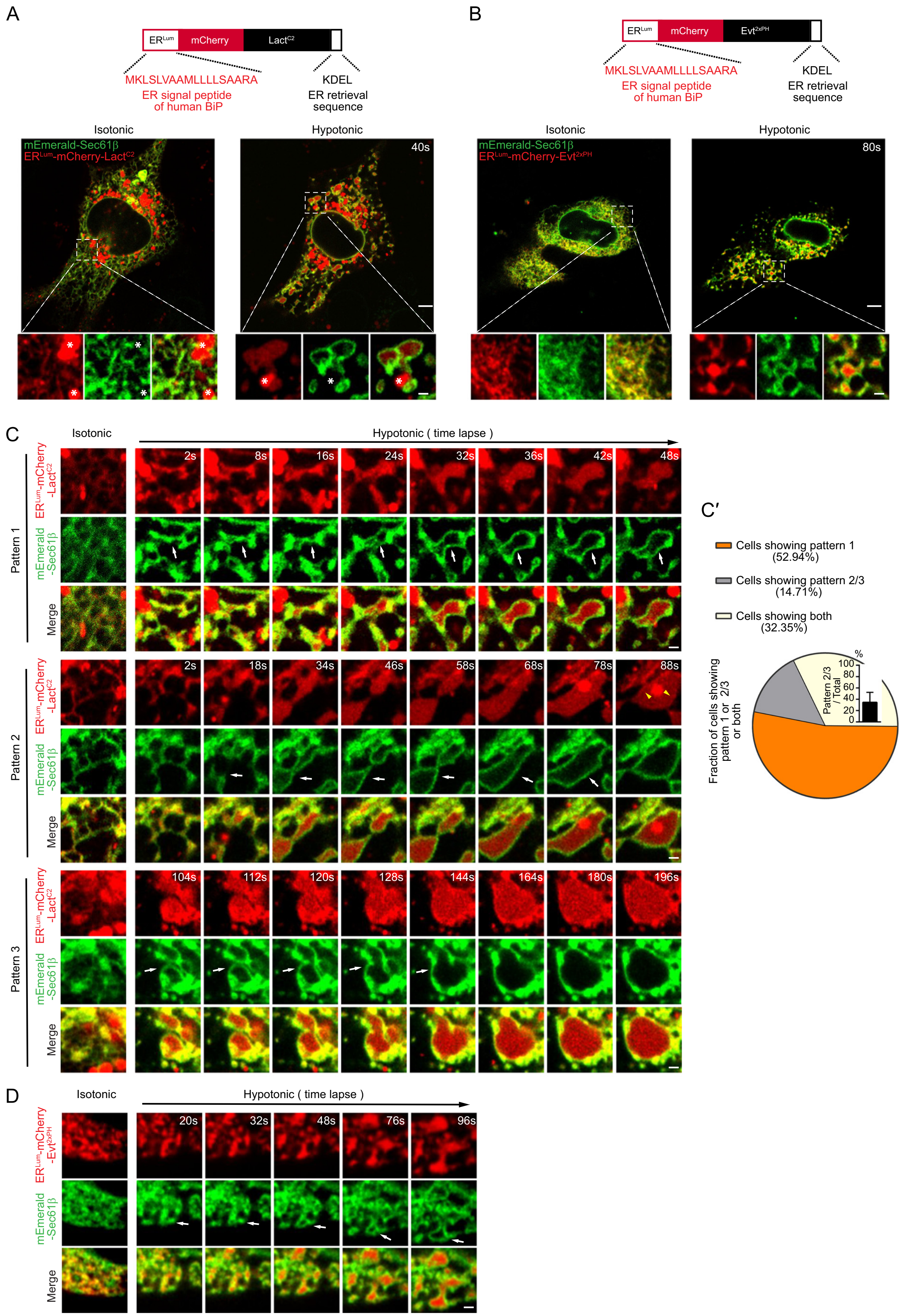

This is a U.S. Government work and not under copyright protection in the US; foreign copyright protection may apply

Figure EV3. Membrane association of PS reporters targeted to the ER lumen after hypoosmotic challenge.

Related to Fig. 4. (**A**, **B**) Cartoon of the design of the ER lumen-targeted PS reporters [ER^{Lum}-mCherry-$Lact^{C2}$ (**A**) or ER^{Lum}-mCherry-Evt^{2xPH} (**B**)] and their localization when expressed in live U2OS cells together with mEmerald-Sec61β. Cells are shown before (left) and after (right) a brief exposure to hypotonic conditions. Inserts show enlarged areas from regions indicated in the whole cell pictures (scale bar, 5 μm). In addition to being in the lumen of ER, the ER^{lum}-mCherry-$Lact^{C2}$ also decorates some non-ER vesicular structures that lack the ER marker mEmerald-Sec61β (marked with white asterisks). Notably, the ER^{Lum}-mCherry-Evt^{2xPH} is only confined to the ER lumen (lower panels, scale bar, 1 μm). (**C**) Time-lapse of confocal images showing the expanding ER structures covering the first 200 s during hypotonic treatment. U2OS cells expressed the ER^{Lum}-mCherry-$Lact^{C2}$ probe together with the mEmerald-Sec61β. Upon ER expansion, ER^{Lum}-mCherry-$Lact^{C2}$ exhibits three typical patterns of distributions: they distribute uniformly within the ER lumen (pattern 1, see also Movie EV2), they show a faint localization to the luminal leaflet of the ER (LER) (pattern 2, see also Movie EV3), or they show moderate but recognizable localization to the LER (pattern 3, see also Movie EV4). White arrows indicate the gradual swelling of the ER lumen. Yellow arrowheads indicate the mild or moderate enrichment of $Lact^{C2}$ in LER. Scale bar, 1 μm. (**C′**) Pie diagram showing the fraction of cells displaying the different patterns of $Lact^{C2}$ localization to the LER. Cells showing pattern 1 (orange, 18/34 cells from 3 independent experiments), cells showing pattern 2 or 3 (gray, 5/34 cells from 3 independent experiments), and cells showing a mix of 1, 2, or 3 (light yellow, 11/34 cells from 3 independent experiments). The insert within the pie diagram indicates the percentage of patterns 2/3 within the group of cells that showed all three distribution patterns of $Lact^{C2}$ (Data shown are mean ± SEM). (**D**) Time-lapse images showing the localization of ER^{Lum}-mCherry-Evt^{2xPH} during a 200 s period after hypotonic treatment. ER^{Lum}-mCherry-Evt^{2xPH} shows uniform distribution in the ER lumen without any sign of membrane localization in all the cells observed (41/41 cells from 3 independent experiments, see also Movie EV5). White arrows indicate the gradual separation of the membrane of the swelling ER. Scale bar, 1 μm.

 This is a U.S. Government work and not under copyright protection in the US; foreign copyright protection may apply

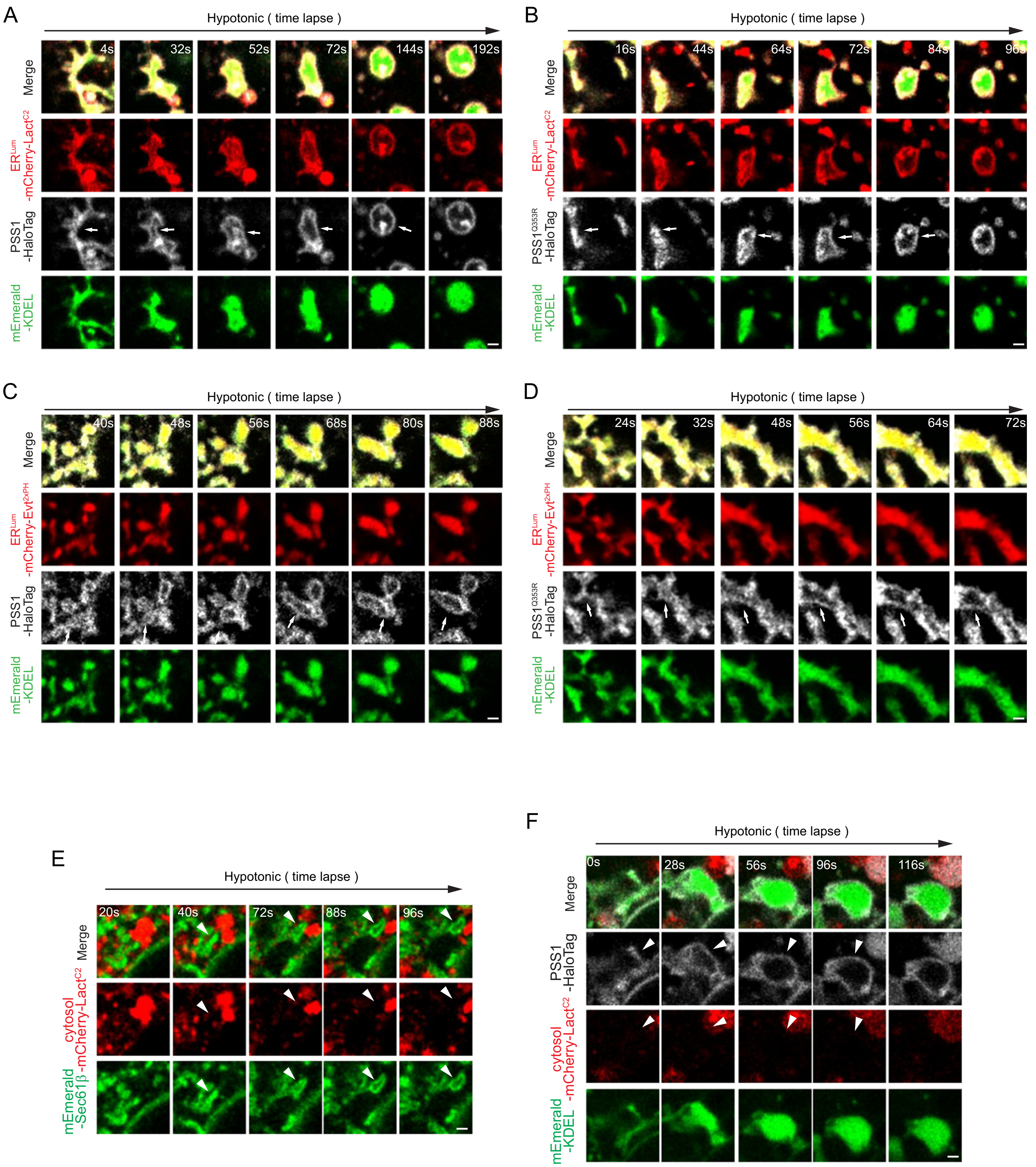

This is a U.S. Government work and not under copyright protection in the US; foreign copyright protection may apply

Figure EV4. Membrane association of PS reporters targeted to the ER lumen in cells producing more PS as assessed after hypoosmotic challenge.

Related to Fig. 4. (**A**, **B**) Live-cell imaging of U2OS cells transiently expressing the ER^{Lum}-mCherry-$Lact^{C2}$, ER luminal marker mEmerald-KDEL, together with PSS1-HaloTag (**A**) or $PSS1^{Q353R}$-HaloTag (**B**) during hypotonic challenge. Enlarged images show the individual channels in a representative cell during hypotonic ER swelling (see also Movies EV6 and 7). Note the clear membrane localization of the $Lact^{C2}$ reporter (red channel) from the luminal leaflet of the ER in all the cells observed (49/49 cells for PSS1-HaloTag and 43/43 cells for $PSS1^{Q353R}$, $n = 3$ independent experiments). Scale bar, 1 μm. (**C**, **D**) Same as in (**A**, **B**) using ER^{Lum}-mCherry-Evt-2x-PH instead of $Lact^{C2}$. (see also Movies EV8 and 9). No membrane localizations are observed with this PS reporter even when PS production is significantly enhanced by the PSS1-Q353R mutant (**D**) in all those cells (45/45 cells for PSS1-HaloTag and 41/41 cells for $PSS1^{Q353R}$, $n = 3$ independent experiments). Scale bar, 1 μm. (**E**, **F**) Live-cell imaging of U2OS cells transiently expressing the cytoplasmic mCherry-$Lact^{C2}$ probe together with mEmerald-Sec61β (**E**), or mEmerald-KDEL. PSS1-HaloTag was also expressed in cells shown in (**F**). Time-lapse of images of the individual channels are shown during a hypotonic challenge. Note that no membrane signal is visible once the probe is facing the cytoplasmic leaflet of the ER membrane (35-35 cells (for **E** and **F**) from 3 independent experiments). Scale bar, 1 μm).

This is a U.S. Government work and not under copyright protection in the US; foreign copyright protection may apply

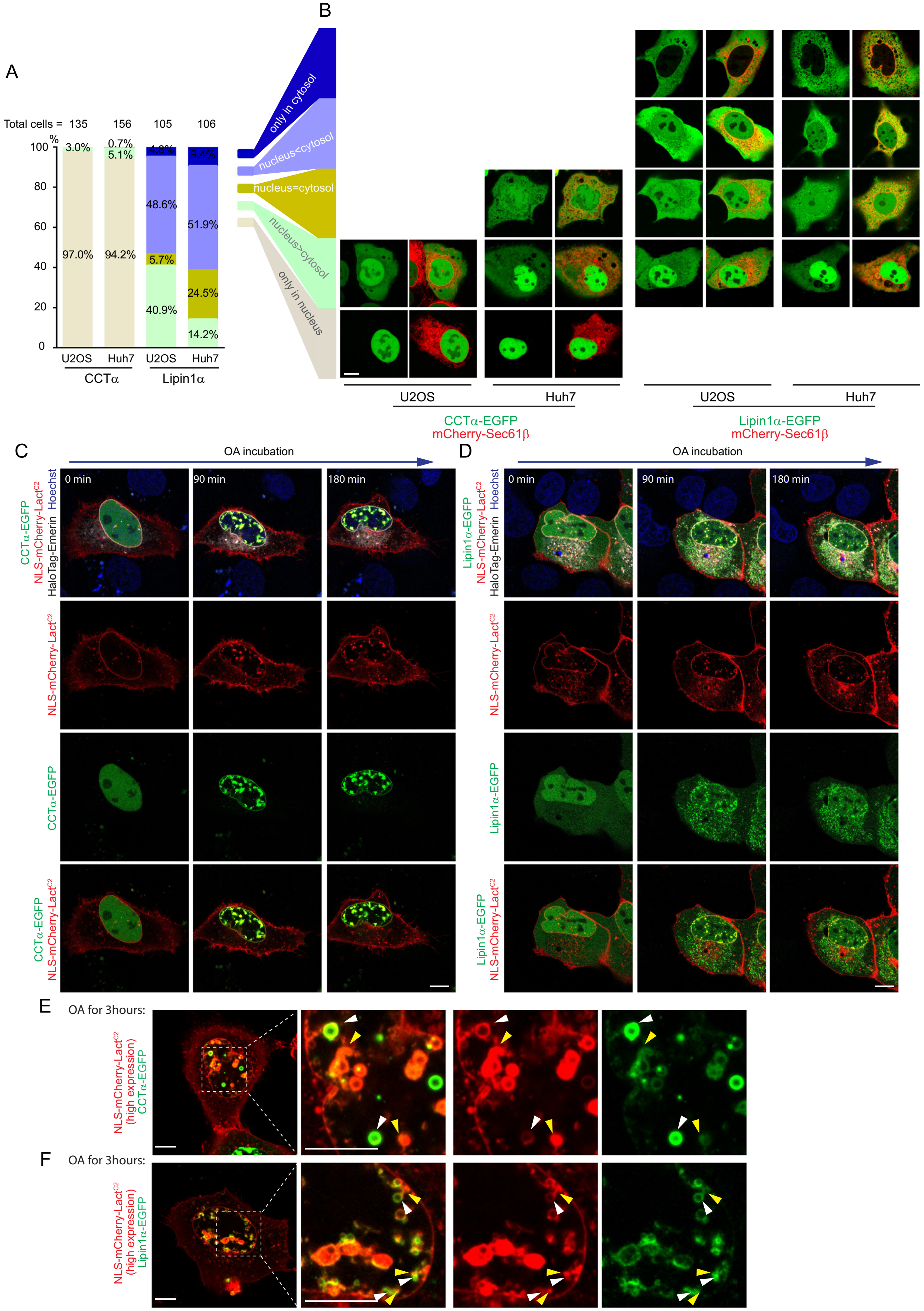
A
Total cells = 135 156 105 106
100 %
80
60
40
20
0
3.0%
97.0%
0.7%
5.1%
94.2%
4.8%
48.6%
5.7%
40.9%
9.4%
51.9%
24.5%
14.2%
U2OS Huh7 U2OS Huh7
CCTα Lipin1α
B
only in cytosol
nucleus<cytosol
nucleus=cytosol
nucleus>cytosol
only in nucleus
U2OS Huh7
CCTα-EGFP
mCherry-Sec61β
U2OS Huh7
Lipin1α-EGFP
mCherry-Sec61β
C
OA incubation
0 min
90 min
180 min
CCTα-EGFP NLS-mCherry-Lact^C2 HaloTag-Emerin Hoechst
NLS-mCherry-Lact^C2
CCTα-EGFP
CCTα-EGFP NLS-mCherry-Lact^C2
D
OA incubation
0 min
90 min
180 min
Lipin1α-EGFP NLS-mCherry-Lact^C2 HaloTag-Emerin Hoechst
NLS-mCherry-Lact^C2
Lipin1α-EGFP
Lipin1α-EGFP NLS-mCherry-Lact^C2
E
OA for 3hours:
NLS-mCherry-Lact^C2 (high expression) CCTα-EGFP
F
OA for 3hours:
NLS-mCherry-Lact^C2 (high expression) Lipin1α-EGFP

Figure EV5. PS plays a role in the INM translocation of CCTα and Lipin1α to the INM and NR in response to oleic acid (OA) treatment.

Related to Fig. 5. (**A**) Distribution of expressed CCTα and Lipin1α between the cytosol and the nucleus in resting U2OS and Huh7 cells. Transiently expressed CCTα-EGFP is primarily localized in the nucleus in almost all the cells in both cell types, whereas Lipin1α-EGFP shows nuclear localization only in a fraction of the cells following a distribution profile shown in examples in (**B**). Scale bar, 10 μm. (**C**, **D**) Live-cell confocal images showing the recruitment of CCTα- or Lipin1α-EGFP to the Lact^{C2}-positive INM and NRs upon loading with oleic acid (OA) in cells expressing low level of NLS-mCherry-Lact^{C2}. Scale bar, 10 μm. (see also Movies EV16 and 17). (**E**, **F**) Live-cell confocal images of cells that show high expression of NLS-mCherry-Lact^{C2}. In such cells, OA loading still causes INM and NR recruitment of CCTα-EGFP or Lipin1α-EGFP (white arrows) but the high Lact^{C2} interferes with the process preventing some NR areas to attract the EGFP-tagged enzymes (yellow arrows). Scale bar, 10 μm.

This is a U.S. Government work and not under copyright protection in the US; foreign copyright protection may apply
